# Is chloroquine chemoprophylaxis still effective to prevent low birth weight? Results of a study in Benin

**DOI:** 10.1186/1475-2875-6-27

**Published:** 2007-03-06

**Authors:** Lise Denoeud, Nadine Fievet, Agnès Aubouy, Paul Ayemonna, Richard Kiniffo, Achille Massougbodji, Michel Cot

**Affiliations:** 1 Mother and Child Health in the Tropics (UR 010), Institut de Recherche pour le Développement, Paris, France; 2 Mother and Child Health in the Tropics, (UR 010), Institut de Recherche pour le Développement, Institut des Sciences Biomédicales Appliquées, Cotonou, Benin; 3 Hôpital de Zone, Ouidah, Benin; 4 Laboratoire de Parasitologie, Faculté des Sciences et de la Santé, Cotonou, Benin

## Abstract

**Background:**

In areas of stable transmission, malaria during pregnancy is associated with severe maternal and foetal outcomes, especially low birth weight (LBW). To prevent these complications, weekly chloroquine (CQ) chemoprophylaxis is now being replaced by intermittent preventive treatment with sulfadoxine-pyrimethamine in West Africa. The prevalence of placental malaria and its burden on LBW were assessed in Benin to evaluate the efficacy of weekly CQ chemoprophylaxis, prior to its replacement by intermittent preventive treatment.

**Methods:**

In two maternity clinics in Ouidah, an observational study was conducted between April 2004 and April 2005. At each delivery, placental blood smears were examined for malaria infection and women were interviewed on their pregnancy history including CQ intake and dosage. CQ was measured in the urine of a sub-sample (n = 166). Multiple logistic and linear regression were used to assess factors associated with LBW and placental malaria.

**Results:**

Among 1090 singleton live births, prevalence of placental malaria and LBW were 16% and 17% respectively. After adjustment, there was a non-significant association between placental malaria and LBW (adjusted OR = 1.43; P = 0.10). Multiple linear regression showed a positive association between placental malaria and decreased birth weight in primigravidae. More than 98% of the women reported regular chemoprophylaxis and CQ was detectable in 99% of urine samples. Protection from LBW was high in women reporting regular CQ prophylaxis, with a strong duration-effect relationship (test for linear trend: P < 0,001).

**Conclusion:**

Despite high parasite resistance and limited effect on placental malaria, a CQ chemoprophylaxis taken at adequate doses showed to be still effective in reducing LBW in Benin.

## Background

In malaria endemic countries, pregnant women are at increased risk of *Plasmodium falciparum *infection [1,2] which leads to increased morbidity and mortality for the mother and her child. In areas of stable transmission, where women have developed acquired malaria immunity, malaria during pregnancy often does not cause symptomatic infection, although it increases the risk for maternal anaemia or death, and low birth weight (LBW) [3,4]. LBW is an important risk factor for infant mortality [5]. It was estimated that around 100,000 infant deaths occur annually in malaria endemic areas in Africa due to pregnancy associated malaria [6]. In these areas, primigravidae have higher prevalence of placental malaria and its associated complications [7,8]. This relates to the property of parasitized erythrocytes to adhere to chondroitin sulphate A (CSA) by means of parasite variant surface antigens (VSA) expressed on the surface of infected erythrocytes. These induce cytoadherence inhibiting VSA_CSA_-specific antibodies which increase in prevalence with increasing parity [9,10].

To prevent the adverse effects of malaria during pregnancy, the World Health Organization (WHO) and most African governments including Benin recommended a weekly chloroquine (CQ) chemoprophylaxis during pregnancy (300 mg per week) until the mid 1990s. Because of the poor compliance of women and the increasing rates of *P. falciparum *resistance to CQ [11], the WHO changed its recommendation to intermittent preventive treatment (IPT), which involves the administration of treatment doses of an antimalarial drug combination (sulfadoxine-pyrimethamine [SP]), given during monthly antenatal care (ANC) visits twice in pregnancy during the second and third trimesters. The efficacy of SP IPT has been studied mostly in East Africa, and has shown reduced prevalence of severe maternal anaemia and LBW [12,13]. However, resistance to SP has been increasing, especially in West Africa and there is a need to evaluate the sue of alternative antimalarial drugs [14].

The Beninese government has recommended SP IPT for pregnant women since the end of 2004, but this strategy is only now commencing. Weekly CQ prophylaxis in pregnancy has been official policy for the past 20 years in most of the country, although this intervention had not been evaluated previously in Benin. The aim of this study was to assess current prevalence of placental malaria and LBW in an observational study in Ouidah, in order to estimate the efficacy of weekly CQ chemoprophylaxis in pregnant women.

## Methods

### Study site and population

The study was conducted from 15^th ^April 2004 to 14^th ^April 2005 in the city of Ouidah, a semi-rural town of approximately 38,000 inhabitants in southern Benin, located 30 km west of Cotonou. In this area, malaria transmission is high year round peaking during the rainy seasons, from April to June, and September to October. *In vivo *CQ resistance in children, defined as clinical treatment failure by day 14, was estimated in 2002 to range between 15% to 61.3%, and SP resistance between 3.3% to 45.9%, dependent on area (Beninese National Malaria Control Program, unpublished data). In Ouidah there is a prevalence of 87.5% resistance to CQ and 50% to SP (Aubouy, unpublished data). HIV prevalence in the general population is approximately 2% [15]. Insecticide treated nets (ITN) are widely used, especially among pregnant women who can obtain them at the time of their ANC visits.

The study was performed in the two main city maternity clinics, Kindji and Hopital de Zone (the latter being also a tertiary referral centre). All pregnant women who delivered in either clinic during the study period were prospectively enrolled after providing oral consent. They were excluded if gestation was less than 22 weeks, in order to exclude early abortions. Ethical clearance was obtained from the Beninese Ministry of Health.

### Study design and variables

Midwives collected data using a standardized questionnaire. The following variables were collected : socio-demographic factors (age, place of living, school attendance, marital status), obstetrical past history (gravidity, parity), pregnancy related factors (number of ANC visits, reported anaemia, oedema, high blood pressure -> 140-90 mm Hg-, acute malaria symptoms during the course of pregnancy), malaria prevention measures (chemoprophylaxis and use of bed nets), and infant characteristics (vital status at birth, birth weight, sex, twins, malformation). Information was obtained from women at delivery and validated with data available in ANC records, such as the estimated gestational age, the number and date of ANC visits, complications of pregnancy, and the date of CQ prescription either for prophylaxis or treatment.

LBW was defined as birth weight < 2500 g, prematurity as gestational age < 37 weeks. High ANC attendance was defined as more than three ANC visits during the course of pregnancy.

Midwives prepared thick and thin malaria smears from intervillous blood. Compliance with CQ chemoprophylaxis was assessed using a sample of 166 women whose urine was obtained at delivery for analysis for CQ metabolites. This sample included women delivered between October 2004 and March 2005, seen on the occasion of antenatal visits, who accepted to provide a urine sample to the midwives, regardless to their declared chemoprophylaxis.

### Biological methods

Placental blood smears were obtained from the maternal side of the placenta and stained with Giemsa, and read at the Zone Hospital laboratory by experienced parasitology technicians. Samples were recorded as positive for placental malaria if an asexual-stage *P. falciparum *parasite was detected, after examination of 200 microscope fields. Malaria pigment was also searched in erythrocytes and/or circulating monocytes, as an indirect indicator for active infection. Placental histology was not performed.

A Haskins semi-quantitative colorimetric method, modified by Mount, was used to quantify CQ in urine [16]. The samples colour intensity was read against an optical colour scale (corresponding to the concentrations: 0; 1; 1.5; 3; 6 and 18 μg/ml). A urine CQ concentration of ≥3 μg/ml, measured by this method, has been shown to be consistent with the ingestion of 300 mg CQ within the previous seven days [17]. This threshold was used to indicate good compliance with CQ chemoprophylaxis.

### Statistical analysis

Data were analysed using the SAS software, 8.1 (SAS Institute, Cary, NC). Only live singleton births were studied. After a global description (number and percentage for categorical data, mean and range for quantitative data), multivariate logistic regression and multiple linear regression analyses were performed to study respectively the association of LBW (categorical) and birth weight (quantitative) with placental malaria adjusting for covariates, using a backward stepwise approach. These analyses were performed to answer different questions: logistic regression provided an operational measure of the factors associated with LBW which was the main outcome; multiple linear regression was more powerful to detect weaker associations with birth weight. Two other variables were included in the models: maternity clinic and gravidity. Other variables included were those with P < 0.20 in the univariate analysis.

To assess the efficacy of interventions (CQ chemoprophylaxis and use of bed nets), we performed logistic regression analyses on LBW and placental malaria, adjusted for gravidity and ANC attendance.

In the final models, all interactions between gravidity and other covariates were tested. For significant interactions, results were stratified (primigravidae versus multigravidae); otherwise they were simply adjusted for gravidity.

## Results

### Study population

A total of 1,176 women were enrolled at delivery, 712 in Kindji (60%) and 464 in the Zone Hospital (40%).

To check the exhaustiveness of these data collected by midwives, they were compared to those available in the maternity clinics birth records, and very few deliveries seem to have been missed (less than 50 during the whole study year). Such missing data were related to high risk deliveries (e.g. a woman referred in the maternity for urgent caesarean section, thus not managed by midwives, unable to complete the questionnaire or to collect the placenta).

There were 55 stillbirths (seven from twin births), and 38 sets of twins, thus 1090 live singleton births were analysed. Mother and infant characteristics are detailed in Table 1. The study participants were on average 25.5 years old (range: 13–44), and 25% (n = 267) were primigravidae (mean gravidity = 3, range: 1–12). The median number of ANC visits in the course of pregnancy was four (range: 0–12), and only six women did not attend antenatal facilities. ANC visits were usually monthly, and their number was directly related to the period between the first ANC visit and delivery. Symptoms of acute malaria (fever) during pregnancy were reported in 300 women (28%), although less than 10% of these (29/300) had a blood film and only five of these had *P. falciparum *present (2%). Treatment was usually with CQ, administered orally (72%). Mean gestational age at delivery was 39 weeks (range: 26–43), and 114 infants (11%) were estimated to be premature (< 37 weeks).

**Table 1:** Mother and infant characteristics, singleton live births, Kindji and Hopital de Zone maternity clinics, 2004–2005

Characteristic	n	%	N
General characteristics			
Maternity clinic			1090
Kindji	693	63.6	
Hopital de Zone	397	36.4	
Season at delivery			1090
Rainy season	435	39.9	
Dry season	655	60.1	
Mother characteristics			
Age (years) (quartiles)			1072
≥20	255	23.8	
21–25	373	34.8	
26–30	244	22.7	
> 30	200	18.7	
Place of living			1090
urban	689	63.2	
rural	205	18.8	
unknown (not reported or not localized)	196	18.0	
School attendance			1051
none	527	50.1	
primary	359	34.2	
secondary, superior	165	15.7	
Gravidity			
primigravidae	267	24.5	1090
multigravidae	823	75.5	
Number of ANC visits			
0–3	400	37.2	1076
> 3	676	62.8	
Reported complications during pregnancy			
high blood pressure	56	5.2	1069
legs oedema	91	8.5	1075
anaemia	57	5.3	1068
proteinuria	91	9.2	992
Reported symptoms of acute malaria	300	28.0	1071
Placental malaria	176	16.7	1052
Infant characteristics			
LBW (< 2500 g)	171	15.7	1087
Sex			
female	522	47.9	1090
male	568	52.1	
Malformation*	14	1.5	909

ANC: antenatal care; LBW: low birth weight

N: total number of women

(*) Most reported malformations: surplus finger (n = 4), hypotrophy (n = 2), palate malformation (n = 1), hydrocephaly (n = 1), other polymalformative syndromes

### LBW and placental malaria

**Figure 1:**
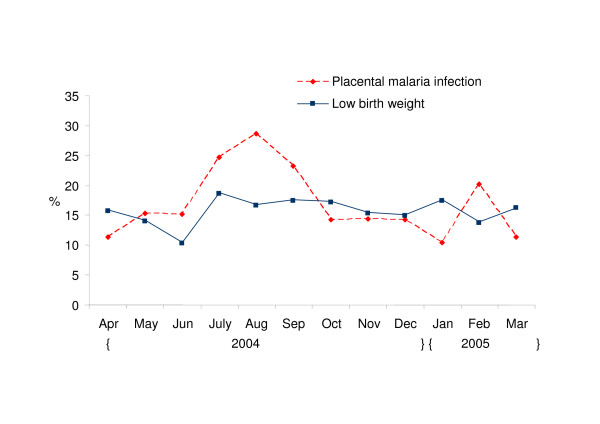
**Monthly variation of low birth weight and placental malaria prevalence in singleton live births**. Ouidah, Kindji and Hopital de Zone maternity clinics, April 2004-April 2005, N = 1090.

Mean birth weight was 2,850 g (range: 800–5,000), and 171 infants (16%) had a LBW (24% in primigravidae and 13% in multigravidae, P < 0,001). The prevalence of placental malaria was 17% (176 of the1052 placental blood samples collected); 27% in primigravidae and 13% in multigravidae (P < 0.001). Monthly variation of LBW and placental malaria prevalence are shown in figure 1. Placental malaria had higher prevalence between July and September 2004 (with main peak of 28.6% in August) and in February 2005 (chi-square goodness-of-fit test comparing the observed monthly distribution to a uniform distribution : P = 0.052). LBW prevalence showed smaller monthly variations (P = 0.82).

Results of the univariate and multivariate logistic regression analyses of factors associated with LBW are presented in Table 2. In univariate analysis, placental malaria was associated with LBW (odds ratio [OR] = 1.76; 95% confidence interval [CI] = 1.18; 2.62). Other unadjusted risk factors for LBW were primigravidae (OR = 2.05; 95% CI = 1.45; 2.90), young maternal age, anaemia, low number of ANC visits and female infant. After adjustment in the multivariate model, placental malaria was not significantly associated with LBW (P = 0.10). An increased LBW risk was observed in primigravidae and with high maternal blood pressure, a low number of ANC visits, or a female infant. Young maternal age was strongly associated with LBW in univariate analysis, but not in the multivariate model after adjustment for gravidae.

**Table 2:** Factors associated with LBW by logistic regression

Characteristic*	**Univariate analysis**		**Multivariate analysis N = 995**	
	Crude OR (95% CI)	P**	Adjusted OR (95% CI)	P**
Placental malaria		0.006		0.10
no	1.00		1.00	
yes	1.76 (1.18–2.62)		1.43 (0.93–2.20)	
Maternity clinic		0.46***		
Kindji	1.00		-	-
Hopital de Zone	0.88 (0.62–1.24)			
Number of ANC visits		0.009		0.03
> 3	1.00		1.00	
≥3	1.56 (1.12–2.17)		1.49 (1.05–2.13)	
Primigravidity		<0.001		<0.001
no	1.00		1.00	
yes	2.05 (1.45–2.90)		2.05 (1.41–2.97)	
Maternal age		0.006***		
≥20	1.00			
[20–25]	0.60 (0.40–0.91)		-	-
[25–30]	0.52 (0.33–0.84)			
> 30	0.45 (0.27–0.76)			
Maternal high blood				
pressure		0.11		0.02
no	1.00		1.00	
yes	1.70 (0.89–3.23)		2.17 (1.10–4.22)	
Maternal anaemia		0.02***		
no	1.00		-	-
yes	2.05 (1.11–3.79)			
Infant Sex		<0.001		0.001
male	1.00		1.00	
female	1.75 (1.26–2.44)		1.79 (1.26–2.56)	

LBW: low birth weight; ANC: antenatal care; OR: odds ratio; CI: confidence interval

(*) Only variables with P value < 0.20 in univariate analysis are shown

(**) P values calculated with the Wald test

(***) Maternity clinic was not included in the model (P > 0.20); anaemia and maternal age did not remain significant in the multivariate model after adjustment

Multiple linear regression showed a clinic effect as birth weight was 72 g [9; 135] higher in the Zone hospital. In this model, gravidity modified the effect of placental malaria infection on birth weight (interaction test: P = 0.02): in primigravidae, placental malaria infection was associated with decreased mean birth weight (regression coefficient = -180 g [-342; -18], P = 0.01), although no association was shown in multigravidae (P = 0.79).

### Antimalarial prevention measures

Self-reported compliance with antimalarial prophylaxis is summarized in Table 3. Seventy-five percent of women (n = 764) reported use of bed nets. All but six reported use of CQ chemoprophylaxis. More than 95% (n = 1,006) declared they had taken an adequate CQ dosage (three 100 mg tablets per week), for a mean duration of five months, commencing at four months gestation.

**Table 3:** Self-reported compliance to antimalarial prophylaxis

Characteristic	n	%
Chemoprophylaxis (N = 1081)		
CQ	1065	98.5
other	10	0.9
none	6	0.6
CQ dosage (tablets per week) (N = 1057)*		
3	1006	95.1
< 3	25	2.4
> 3	26	2.5
Duration of CQ chemoprophylaxis (months) (N = 1040)* (quartiles)		
< 4	246	23.2
[4–5]	348	33.7
[5–7]	257	24.9
≥7	189	18.3
CQ intake during 1^st ^trimester** (N = 1056)*	527	49.9
CQ intake during 2^nd ^trimester** (N = 1044)*	961	92.0
CQ intake during 3^rd ^trimester** (N = 1051)*	1025	97.5
Use of bed nets (N = 1010)	764	75.6

ANC: antenatal care; CQ: chloroquine

(*) The 10 women taking another drug than CQ were not considered here

(**) CQ was considered to be taken during the 1^st^, 2^nd ^or 3^rd ^trimester if the woman said she had taken CQ at least once in the corresponding period.

Of the 166 women whose urine samples were tested for CQ, seven (4.2%) reported fever during the last month of pregnancy, and four (2.4%) had received a curative dose of CQ. These four women were excluded from further analyses. Of the remaining 162 women, only two had no CQ detectable in urine. Low concentrations (< 3 μg/ml) were present in 28% and high concentrations (consistent with the ingestion of 300 mg of CQ within seven days) in 72%.

Table 4 shows the effects of antimalarial prevention measures (prophylaxis and bed nets) on prevalence of placental malaria and LBW. For placental infection, prevention measures (bed nets or CQ) were not found to be protective. These results remained unchanged when considering the presence of malaria pigment in erythrocytes. There was a highly significant association between the duration of chemoprophylaxis and the occurrence of LBW (adjusted OR = 4.61; 95% CI = 2.27; 9.36 for the comparison between the lowest and highest duration quartiles), and a significant duration-effect relationship (chi-square for linear trend: P < 0.001).

**Table 4:** Effects of the use of bed nets and CQ on placental malaria and LBW

	Placental malaria			LBW		
	%	AOR* (95% CI)	P**	%	AOR* (95% CI)	P**

Use of bed nets			0.16			0.45
no	21.7	1.00		19.1	1.00	
yes	15.4	0.76 (0.51–1.11)		14.2	0.86 (0.58–1.27)	
Reported duration of CQ chemoprophylaxis (months)			0.33			<0.001***
< 4	18.0	1.57 (0.82–3.00)		23.1	3.96 (1.9–8.28)	
[4–5]	19.2	1.69 (0.97–2.97)		18.0	2.93 (1.50–5.75)	
[5–7]	15.7	1.37 (0.77–2.44)		9.8	1.52 (0.74–3.13)	
≥7	11.7	1.00		6.5	1.00	
Reported CQ intake during 1^st ^trimester			0.43			0.35
no	16.4	1.00		16.6	1.00	
yes	16.9	1.16 (0.80–1.70)		14.6	1.20 (0.82–1.76)	
Reported CQ intake during 2^nd ^trimester			0.72			0.37
no	19.0	1.00		15.7	1.00	
yes	16.5	0.89 (0.48–1.68)		15.1	1.35 (0.70–2.59)	
Reported CQ intake during 3^rd ^trimester			0.67			<0.001
no	21.7	1.00		46.2	1.00	
yes	16.5	0.80 (0.28–2.26)		14.2	0.24 (0.11–0.56)	

LBW: low birth weight; ANC: antenatal care; AOR: adjusted odds ratio; CI: confidence interval; CQ: chloroquine

(*) Odds ratios adjusted for primigravidity and number of ANC visits

(**) P values calculated with the Wald test

(***) Univariate Chi-square test for linear trend: P < 0,001

Prophylaxis reported to be taken during the last trimester showed a protective effect on LBW (P < 0,001).

## Discussion

In a one-year observation of deliveries in Ouidah, where women were systematically prescribed weekly CQ chemoprophylaxis, CQ was still effective in reducing LBW despite its limited effect on placental malaria.

Placental malaria has been often described as a major cause of LBW [18], but most studies did not adjust for other risk factors. In the present study, we performed multivariate analyses and showed a borderline effect of placental malaria on LBW (P = 0.10). However, in primigravidae, mean birth weight was significantly lower with malaria infection (P = 0.01, multilinear regression), thus confirming that this sub-group is at higher risk for the adverse consequences of gestational malaria.

Female infant sex and high maternal blood pressure are associated risk factors for LBW [19], as well as poor ANC attendance [20]. The latter may reflect different interventions, including standard obstetrical care, malaria prevention and nutritional advice, or a combination of these factors.

In spite of fluctuations in the transmission of malaria, there was no clear effect of the rainy season on the occurrence of LBW, probably because malaria acts as one of the multiple determinants of LBW (like nutritional status [21,22] that may also interfere with foetal growth).

Maternal HIV status and nutritional factors, which may have an influence on LBW [19,21,23], were not available, although where HIV prevalence does not exceed 2% its influence in LBW will be small [14].

In this Beninese population, there was a high ANC attendance and frequent use of bed nets. Compliance with antimalarial prophylaxis was high as 98% of women declared to have taken CQ chemoprophylaxis, during the five months preceding delivery, and most with adequate dosage. This was consistent with the CQ assay in urine in which 99% had detectable CQ. In other African settings, self-reported compliance with prophylaxis has been associated with low levels (< 20%) of amino-quinolines in urine [24]. This was not the case in Benin, where all indicators showed good compliance. The WHO [25] had already pointed Benin as one of the African countries where ANC attendance was the highest and the earliest in the course of pregnancy, with one of the highest coverages for chemoprophylaxis and use of bed nets among pregnant women.

An interesting and unexpected result was that despite low CQ anti-parasite efficacy (nearly 90% in vivo resistance measured in children in Ouidah in 2005, Aubouy, unpublished data), CQ chemoprophylaxis showed protective efficacy for LBW, with a significant duration-effect relationship as the longer the women took chemoprophylaxis, the lower the risk of LBW. This effect was most likely to be associated with chemoprophylaxis, and not with the number of ANC visits, since the analysis was adjusted for this factor. Similarly, there was no differential effect of chemoprophylaxis on placental malaria or LBW when comparing primigravidae and multigravidae.

Parasite resistance to chloroquine is considered to relate to reduced efficacy in terms of birth weight and maternal anaemia. It is one of the main reasons why CQ has been replaced by intermittent SP in malaria prevention policies in pregnancy [26]. However, in the 1990's, when resistance to CQ was developing in Western Africa, it was shown that with moderate resistance there was still good protection of the foetus from the main consequences of placental malaria [27]. The mechanism by which placental malaria leads to LBW is not clearly understood. This association was shown to be parasite density-dependent, with higher risks for LBW in women with high parasitemias [21], indicating that parasite accumulation in the placenta was contributory. Activity of CQ on residual sensitive *P. falciparum *strains could lead to a partial reduction in placental parasitemia, and decrease of LBW.

In addition to parasite resistance, another reason for abandoning CQ prophylaxis in pregnant women in sub-Saharan Africa is the reported poor compliance in pregnancy [28]. This is not true for Beninese women, at least in our study area, who showed a good adherence to prophylaxis and also at adequate dosage. The combined effect of these two factors (partial antimalarial activity of CQ and good compliance) is probably the explanation for the discrepancy between presumed *P. falciparum *resistance to CQ and a protective effect for reducing risk of LBW.

## Conclusion

From a public health point of view, these results and previous studies [27] indicate that there may be an important time interval between the onset of *P. falciparum *resistance to CQ and the subsequent development of adverse consequences to the foetus health. Although a return to CQ prophylaxis in Africa cannot be seriously considered, these findings could be relevant to SP IPT prevention policy. As resistance to SP is rapidly spreading in endemic areas, it is essential to find alternative drugs for IPT. However, residual activity of SP may also be sufficient to maintain benefits to the foetus while alternative antimalarials are evaluated.

## Authors' contributions

LD participated in the design of the study and the collection of data, performed the statistical analysis and drafted the manuscript. NF and AA participated in the design and coordination of the study, the collection of data in the field and made helpful comments on the manuscript. PA, RK and AM participated in the coordination and design of the study and revised the paper critically. MC was the conceptor of the study, participated to its design and coordination, helped to draft the manuscript and revised it. All authors read and approved the final manuscript.
